# Bisphenol A and Metabolic Diseases: Challenges for Occupational Medicine

**DOI:** 10.3390/ijerph14090959

**Published:** 2017-08-25

**Authors:** Lidia Caporossi, Bruno Papaleo

**Affiliations:** INAIL—National Institute for Insurance against Accidents at Work—Department of Occupational and Environmental Medicine, Epidemiology and Hygiene, Laboratory of Health Surveillance and Health Promotion, 00078 Monte Porzio Catone, Roma, Italy; b.papaleo@inail.it

**Keywords:** bisphenol A, obesity, diabetes, workers

## Abstract

The prevalence of metabolic diseases has markedly increased worldwide during the last few decades. Lifestyle factors (physical activity, energy-dense diets), together with a genetic predisposition, are well known factors in the pathophysiology of health problems. Bisphenol A (BPA) is a chemical compound used for polycarbonate plastics, food containers, epoxy resins coating metallic cans for food and beverage conservation. The ability of BPA to act as an endocrine disruptor—xenoestrogen in particular—is largely documented in literature, with numerous publications of in vivo and in vitro studies as well as epidemiological data on humans. Recently, different researchers studied the involvement of BPA in the development of insulin resistance; evidences in this way showed a potential role in etiology of metabolic disease, both for children and for adults. We review the epidemiological literature in the relation between BPA exposure and the risk of metabolic diseases in adults, with a focus on occupational exposure. Considering published data and the role of occupational physicians in promoting Workers’ Health, specific situations of exposure to BPA in workplace are described, and proposals for action to be taken are suggested. The comparison of the studies showed that exposure levels were higher in workers than in the general population, even if, sometimes, the measurement units used did not permit rapid comprehension. Nevertheless, occupational medicine focus on reproductive effects and not metabolic ones.

## 1. Introduction

The incidence of metabolic syndromes, obesity and type 2 diabetes in particular, has steadily increased worldwide over the past 30 years; the cause of this situation most certainly lies in high calorie diets and ever less movement, besides possible genetic predisposition. Some data show that exposure to endocrine disruptor chemical compounds, which are ubiquitous at an environmental and/or food level, can play a role in the development of these diseases [1,2,3]. Among these substances, Bisphenol A (BPA) has aroused particular interest for its presence in many everyday products, as component in polycarbonate plastics and epoxy resins, and the possibility of contamination of food with which it comes into contact; this situation is verified by noticeable exposure to the general population [4].

Table 1 shows the most relevant sources of potential exposure to BPA in living environments.

In the literature, several investigations have tried to clarify the mechanisms of interaction between BPA and metabolic syndromes: in vitro studies have supported the theory that exposure to BPA, in particular, moments of the development of adipose tissue, can cause metabolic dysfunction of the adipocytes and inflammation, which may lead to an increase of conditions correlated with obesity [7]. In the case of exposure to several endocrine disrupters simultaneously (phthalates, organotin and BPA), the effects could be very different, not necessarily in terms of additivity [8]. Moreover, important evidence shows the ability of BPA to act as xenoestrogens binding to nuclear and non-nuclear membrane estrogen receptors, even interfering with hormone synthesis and causing epigenetic deregulation [9].

In vivo investigations show how exposure to BPA in the first days of life causes a weight increase [10], particularly in female rats; researchers also hypothesize a change in energy metabolism suggesting alterations in neurotransmitter signals [11]. Nevertheless, there is still some disagreement over these results [12].

It is well known that with regards to the role of estrogens in pancreatic cells, as regulators of organ functionality through estrogen receptors (essentially estrogen receptor α ), low estrogen levels are associated with a glucose intolerance and the development of insulin resistance [13]. BPA as a xenoestrogen, is capable of mimicking the action of estrogens on a pancreatic level by inducing a positive or negative change in insulin production, similar to 17β-estradiol [14,15,16]. Exposure of the liver to BPA can lead to glucose production and a reduction of glycogen synthesis with reduction of glucose oxidation and damage to insulin signals, while at a muscle level, BPA is able to reduce the use of glucose and insulin sensitivity [17].

During studies on humans, the main focus was on potential exposure for children, also because of the emerging issue of childhood obesity [18]: surveys on school-aged children [19] have highlighted how urinary levels of BPA were directly related to body mass index (BMI), with greater significance in those aged between 8 and 11, and particularly in females. For urinary levels of BPA > 2 µg/L a risk factor has been calculated twice as high for females above the 90th percentile of weight at 9–12 years of age (Odd ratio OR = 2.32, 95% with a confidence interval = CI 1.15–4.65) [20]. Similar data are confirmed [21] through a significant correlation between being overweight and obese and BPA urinary levels, in comparison with dosages in children of normal weight. BPA may also have a role in the onset of insulin resistance in children, particularly among obese children [22]. 

Possible evidence of prenatal exposure to BPA in the future development of adipocytes, and therefore potential metabolic disorders in children has been investigated, but studies are very contradictory, with negative [23,24] and positive evidences [25,26].

In workplaces, the preparation and the production of manufactured articles containing BPA can lead to higher exposure levels for workers, with respect to that found in living environments. The productive sectors in which BPA is used are: the petrochemical industry, in the synthesis of the product; the plastics industry, in the production of polycarbonate plastics; the paints and resins industry, in the production of epoxy-based products; the food canning industry, in plastic coatings; as well as activities in which these products are being used for specific purposes (e.g., the application of epoxy resins as surface insulation or in epoxy paints) [27].

This study is a reflection on the potential risk of the metabolic disorders in the categories of workers potentially exposed to BPA, and provides helpful information for occupational physicians in directing their health checks.

## 2. Materials and Methods

A bibliographical collection was made using PubMed and Scopus search engines and using as search items: “BPA and diabetes” “BPA and obesity” “BPA and metabolic disorders”, “BPA and workers” “BPA and occupational exposure”; surveys prior to 2002 and articles not available in English, Spanish, or Italian were excluded from the study. Epidemiological surveys on adult men and women have been taken into consideration for the review in question.

**Figure 1:**
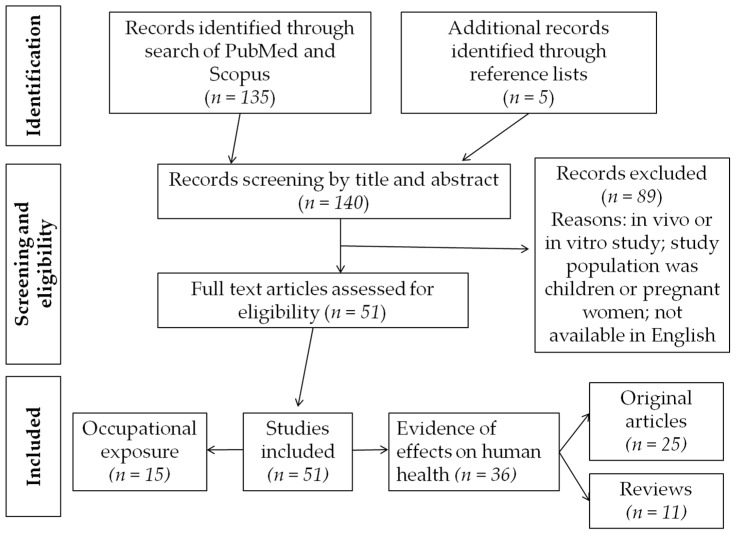
Descriptive diagram of the method used for bibliographical selection of articles.

The descriptive diagram of the selection of articles carried out is shown in Figure 1.

## 3. Evidence of the Effects of Metabolic Type of Exposure to Bisphenol A on Health

### 3.1. Population Studies

Bisphenol A is an endocrine disruptor rapidly assimilated by the human organism without bioaccumulation, but its ubiquitous use actually causes a daily exposure on different levels [28]. Currently available epidemiological data suggest that BPA can act negatively on metabolic homeostasis, exacerbating or accelerating the development of obesity, metabolic syndrome, and type 2 diabetes [28].

A brief overview of the original articles analyzed is shown in Table 2.

The majority of epidemiological studies present in the literature are cross-sectional studies, and this reduces, to a certain extent, the predictive power of the datum [53]. Overall, published data certainly suggest a link between environmental exposure to BPA and an increase of metabolic disorders such as obesity, through the alteration of adipocyte differentiation, some cardiovascular disorders [54], type 2 diabetes, interference with different cellular communication routes involved in glucose homeostasis, and the onset of insulin resistance [55]. Nevertheless, several authors [4,55,56,57,58] underline the opportunity to produce prospective cohort studies, with particular attention to confounding factors such as high-calorie diet and life habits, but also genetic variables and comorbidity. Studies of dose–response relationship, with the characterization of exposure, are required in order to be able to extrapolate data of greater force and predictive significance, especially to capture causality evidence [4,58]. Studies on the data of the National Health and Nutrition Examination Survey (NHANES) [35,37,43,48] are solid from the methodological point of view, and propose an important number of samples; nevertheless, cross-sectional study characteristics severely restrict the possibility for extrapolating elements of causality, i.e., the correlation between levels of BPA and BMI.

The characterization of the exposure is central, because, for example, in the case of obesity, eating habits involving a wide consumption of canned foods or packaged foods with a high caloric content can be regarded as food more easily “contaminated” by BPA, becoming an important confounding factor in the study [59]. Sometimes, there are methodological limitations in the studies published that make the conclusions of lesser impact; for example, an influencing factor can be the choice of the biological indicator to characterize the magnitude of exposure to BPA: in some cases urinary BPA (combined or total) is proposed as a simple concentration, on a spot urine sample, without proceeding with a standardization for the grams of urinary creatinine, and this choice implies a very limited interpretation of the datum [59].

### 3.2. The Workplace: Epidemiological and Exposure Data

The surveys aimed at assessing occupational exposure to BPA often focus on the exposure data and their interpretation. Sometimes, surveys limit the considerations relating to potential effects on health. Any adverse effects considered in the literature have mainly focused on the ability of BPA to act as an endocrine disruptor: particularly for male or female reproductive functions, and altered levels of thyroid and sex hormones. This outlook is understandable in view of the sensitivity of the reproductive sphere, which greatly affects the quality of life of individuals and of the evidence of the literature with respect to the capacity of BPA to act as xenoestrogen. Some authors emphasize the possibility of dermal exposure related to contact with thermal papers (such as by cashiers), which is another aspect of certain interest and which might require additional considerations with respect to possible exposure routes [60].

A description of the studies conducted in the workplace is shown in Table 3.

## 4. Discussion

The selected articles show exposure levels in the general population were considerably higher than among occupational exposed workers, as predicted. This aspect must be taken in consideration depending on the nation in which the studies were done, as some were made in developing countries (i.e., Nigeria), or in countries (i.e., China) where the regulations for the protection of workers from chemicals have lower standards compared to Western countries. It should be noted how the studies conducted in workplaces focused almost exclusively on the assessment of endocrine effects. The possibility of investigating correlations between exposure to BPA and the presence of diabetes or obesity are absent from occupational medicine research.

The role of the occupational physician has changed over the years, from the prevention of occupational risks and health monitoring, to health promotion in general, thus integrating the concept of “wellbeing” in the widest possible way. In this perspective, considerations regarding unnecessary and harmful habits such as tobacco, or incorrect dietary habits, have become fields of intervention for the occupational physician.

These additional tasks introduced by law, could be integrated with the risk assessment of BPA, and moreover evaluate the very different and often competing causes of metabolic diseases, starting from genetic predisposition, eating and life habits.

From the assessment of exposure levels recorded in workplaces (Table 3) and comparison with the values recorded in population groups (Table 2), a concern is raised: a correlation between BPA exposure and diabetes or obesity, as highlighted in the population studies, may be found, with greater probability, in professionally exposed workers.

Related to these data, it is required that occupational physicians define health surveillance protocols, in consideration of the potential metabolic disorders that might arise, from a diagnostics point of view and for the evaluation of different possible risk sources.

## 5. Conclusions

Genetic predisposition and environmental factors, particularly rich nutrition and physical inactivity, play a key role in the onset of metabolic disorders; nevertheless they may not fully clarify the extent of the increase in this type of pathology over the last century. The United States Center for Disease Control and Prevention (CDC) has documented how the occurrence of diabetes has increased from 0.93% in 1958 to 6.29% in 2008, and the increase in obesity (BMI ≥ 30) in the adult population in the United States from 13.4% in 1960–1962 to 35.1% in 2005–2006 [74]. These data require particular attention from the scientific community to identify other exposure factors which may follow this trend [75].

A recent study [76], investigated health costs that could place a burden on public finances in the European Union due to obesity and diabetes, caused exclusively by endocrine disruptors; the estimate concerning BPA was of a 20–69% probability that a prenatal exposure to BPA can cause 42,400 obese children, with an associated cost throughout their life of 1.54 billion euro.

In view of the data submitted, with regard to the population surveys, significant elements of a correlation between exposure to BPA and metabolic disorders are revealed. However, the definition of prospective studies to improve the predictability of data and their statistical force is desirable. The use of standardized measurement units is recommended (i.e., for the micrograms of urinary creatinine in the case of urine); this would allow data comparability and correct exposure assessment, taking in consideration the matrix of analysis.

The presence of metabolic syndrome whose etiology might be an occupational exposure to BPA seems to be a real possibility that the occupational physician must consider in his working activity, to protect the Workers’ Health.

On the other hand, data from literature, based on the general adult and worker population, call for epidemiological investigations aimed at assessing the possible correlation between occupational exposure to BPA and metabolic disorders, with specific regard to obesity and type 2 diabetes in workplaces, to further direct the activities of the occupational physician towards safeguarding the health of workers.

## Figures and Tables

**Table 1:** Main sources of exposure to Bisphenol A (BPA) and levels found [5,6].

Sources of Contamination from BPA	Concentration of BPA (Range)
Aquatic environment	8.000–21.000 ng/L
Air	0.002–0.208 ng/L
Dust	800–10.000 ng/g
Thermal paper	54.000–79.000 ng/cm^2^
Meats	17–602 ng/g
Fish	5–109 ng/g
Vegetables and fruits	9–76 ng/g
Beverages	1–18 ng/g
Dairy products	21–43 ng/g
Infant formula	0.1–13 ng/g
Cans	2–82 ng/g
Plastics	0.2–26 ng/g
Dental materials	13.000–30.000 ng

**Table 2:** General description of results from population studies.

Focus	Study Type	Number of Subjects	Population Type	Average BPA Concentration	Results	Ref.
Diabetes	Cross-sectional	3516	Prediabetes subjects (glucose: 100–125 mg/dL) older than 20	1.93–2.22 ^a^	Independent of traditional diabetes, risk factors higher, urinary BPA levels were found to be associated with prediabetes situation.	[28]
Diabetes/cardiovascular disease/obesity	Cross-sectional	1455	adults aged 18–74	4.5–4.7 ^a^	Positive correlation between urinary BPA levels and increased diagnosis of cardiovascular dosease, type 2 diabetes, but not BMI.	[29]
Type 2 diabetes	Cross-sectional	3967	Adults older than 20	3.9–4.0 ^a^	Increased type 2 diabetes was significantly associated with higer urinary levels of BPA.	[30]
Diabetes/obesity	Crosssectional	296	Reproductive aged women between 30–49	1.38 ^c^	Urinary BPA levels were positively correlated with BMI, waist circumference, and insulin resistance.	[31]
Type 2 diabetes	Cross-sectional	3423	Adults older than 40	0.8 ^a^	A weak association was found between urinary BPA levels and increased diabetes.	[32]
Type 2 diabetes	Cross-sectional	1210	Adults older than 40	2.1 ^a^	A weak association was found between urinary BPA levels and increased diabetes.	[33]
Diabetes/cardiovascular disease/liver function	Cross-sectional	2948	Adults aged 18–74	1.8–2.5 ^a^	Positive correlation between urinary BPA levels and increased diagnosis of cardiovascular disease, type 2 diabetes and liver enzymes, but with fewer associations in most recent data.	[34]
Inflammatory markers/obesity/diabetes	Cross-sectional	76	Male aged 47–59	1.04 ^d^	Data support the BPA role in visceral obesity-related low grade chronic inflammation.	[35]
Type 2 diabetes	Cross-sectional	4389	Adults older than 20	2.0 ^a^	Higher urinary BPA levels were significantly and positively associated with incidence of type 2 diabetes and hemoglobin A1c.	[36]
Cardiovascular disease	Cross-sectional	591	Subjects with and without CAD	1.3–1.5 ^b,a^	Compared to controls, people with CAD had shown significantly higher urinary BPA levels.	[37]
Cardiovascular disease	Case/control	1619	Adults aged 40–74 with or without CAD	1.2–1.4 ^a^	Higher incident of CAD during 10.8 years of follow-up was positively associated with higher urinary BPA levels.	[38]
Obesity	Prospective cohort	977	Adults older than 40	0.8–5.0 ^a,b^	Weak associationbetween BPA levels and greater weight.	[39]
Cardiovascular disease	Cross-sectional	745	Adults older than 40	2.3 ^a^	Positive association between prevalence of peripheral arterial disease and BPA levels in urine.	[40]
Cardiovascular disease	Cross-sectional	521	Adults older than 60	1.2 ^c^	Positive association of reduced heart rate variability and increased hypertension with urinary levels of BPA.	[41]
Obesity	Cross-sectional	2747	Adults aged 18–74	2.1 ^c^	Higher urinary BPA was significantly associated with higher BMI and waist circumference.	[42]
Obesity/hormones	Prospective cohort	890	Adults older than 70	2.1–3.9 ^d^	No significant relationship between BPA levels and indices of at mass or fat distribution were found.	[43]
Obesity/type 2 diabetes	Cross-sectional	3390	Adults older than 40	0.8 ^a^	Higher urinary BPA was significantly associated with higher BMI, abdominal obesity, and insulin resistance.	[44]
Obesity	Cross-sectional	223	Adults older than 18	2.85 ^c^	Weak positive association between urinary BPA levels and BMI.	[45]
Obesity/sex hormone concentrations	Cross-sectional	282	Healthy premenopausal, non–obese women aged 20–55	2.3 ^a^	Positive association between body weight, BMI, fat mass, and serum leptin concentrations with urinary BPA levels.	[46]
Obesity	Cross-sectional	3967	Adults older than 20	3.9–4.0 ^a^	Higher urinary BPA was significantly associated with higher BMI and waist circumference.	[47]
Obesity	Cross-sectional	85	Female aged 16–58	1.5–1.7 ^a^	Positively significant correlation between BMI and BPA, cholesterol, LDL-c and leptin; while a negative correlation between BMI and adiponectin and HDL-c.	[48]
Obesity	Cross-sectional	82	Men and women with subfertility	1.3 ^a^	None association between BPA levels and BMI.	[49]
Obesity/sex hormones/PCOS	Case/control	73	Women with and without PCOS, obese and not	0.7–1.2 ^d^	Positive association between increased serum BPA, BMI, and sex hormone concentrations.	[50]
Diabetes/sex hormones/PCOS	Cross-sectional	171	Women with and without PCOS, obese and not	0.7–1.1 ^d^	Positive association between increased serum BPA and sex hormone concentrations. BPA was positively correlated with insulin resistance.	[51]
Type 2 diabetes/PCOS/inflammation	Cross-sectional	60	Lean and obese women with and without PCOS, aged 23–33	0.1–0.7 ^d^	Women with higher levels of serum BPA had more severe insulin resistance, increased markers of chronic inflammation. Women with PCOS had higher serum BPA levels than controls.	[52]

^a^ µg/L, unadjusted urinary BPA; ^b^ median; ^c^ µg/g, urinary BPA adjusted for creatinine; ^d^ µg/L, serum BPA. BPA: Bisphenol A; BMI: body mass index; CAD: coronary artery disease; LDL: low density lipoprotein; HDL: high density lipoprotein; PCOS: polycystic ovary syndrome.

**Table 3:** General description of the studies conducted on workers with occupational BPA exposure.

Focus	Study Type	Number of Subjects	Population Type	Average BPA Concentration (Subject or Controls-Cases)	Results	Ref.
Urinary biomonitoring	Case/control	90 cases/44 controls	Cashiers exposed by thermal paper (dermal exposure) and not.	2.89 ^c^–6.76 ^c^	A significant increase in urinary total BPA concentration was observed for cashiers handling daily thermal paper receipts.	[59]
Urinary biomonitoring	Case/control	108 cases/88 controls	Workers of a plastic industry and not	25.10 ^a^–43.88 ^a^	There was significant increase in the mean urinary BPA output by industry workers, especially male;, those who had spent ≥6 years in the industry showed a significant increase in BPA output compared to those who spent <6 years.	[60]
Urinary biomonitoring and laboratory abnormalities	Cross-sectional	28	Workers in two semiautomatic epoxy resin factories	31.96 ^b,c^	Higher BPA concentrations were associated with clinically abnormal concentrations of FT3,FT4,TT3,TT4,TSH, glutamic-oxaloacetic transaminase and, γ-glutamyl transferase.	[61]
Male sexual dysfunction	Case/control	230 cases/404 controls	Workers of BPA manufacturer and epoxy resin manufacturers and not.	1.2 ^c^–57.9 ^c^	Exposed workers had a statistically increased risk of erectile difficulty (OR = 4.5, 95% CI 2.1–9.8) and ejaculation difficulty (OR = 7.1, 95% CI 2.9–17.6).	[62]
Urinary biomonitoring and reproductive hormones	Case/control	106 cases/250 controls	Female workers from manufacturers of epoxy resin	0.9 ^b,c^–22.2 ^b,c^	A significant positive association was found between urine BPA level and serum prolactin and progesterone concentration.	[63]
Urinary biomonitoring and reproductive hormones	Cross-sectional	592	Male workers in industry	685.9 ^c,d^	Males, whose urine BPA level was in the second, third, and highest quartiles had respectively 1.58-, 1.33- and 3.09-fold increased prevalence of having high prolactin levels, and the highest quartile was associated with 1.63- and 1.50-fold increased prevalence of having elevated estradiol and sex hormone-binding globulin levels.	[64]
Serum biomonitoring and reproductive function	Case/control	281 cases/278 controls	Workers occupationally exposed to BPA	0.0 ^e^–18.75 ^e^	Increased serum BPA level was associated with decreased mean serum androstenedione level (0.18 ng/mL, 95% CI 0.22–0.13) and increased mean serum SHBG level (2.79 nmol/L 95%, CI 2.11–3.46).	[65]
Serum biomonitoring and sex hormone levels	Cross-sectional	290	Male workers, with and without BPA exposure	0.276 ^d,e^–3.198 ^d,e^	Increasing serum BPA concentration was statistically associated with decreased androstenedione levels, free testosterone levels, free androgen index, and increased sex hormone binding globulin levels.	[66]
Urinary biomonitoring and reproductive function	Cross-sectional	427	Male workers in BPA and epoxy resin industry, exposed and not to BPA	1.2 ^c,d^–53.7 ^c,d^	Increasing urine BPA level was associated with more difficulty having an erection and lower ejaculation strength.	[67]
Urinary and serum Biomonitoring	Cross-sectional	952	Workers of industrial factories and family members	24.93 ^c^ and 2.84 ^e^	Half of the study subjects had detectable BPA in their urine samples, BPA levels were influenced by gender and smoking status.	[68]
Urinary biomonitoring and reproductive function	Case/control	42 exposed male workers and 42 controls	Workers whose job was to spray epoxy resin	0.52 ^d,f^–1.06 ^d,f^	Results suggest that bisphenol A may disrupt secretion of gonadotrophic hormones in men	[69]
Serum biomonitoring and polycystic ovary syndrome (PCOS)	Case/control	62 PCOS women and 62 controls	PCOS women, working as market seller and healthly women	0.16 ^e^–0.48 ^e^	In BPA-exposed PCOS women, BPA level was higher than healthy women, together with higher levels of triglyceride, cholesteriol, TSH and LH:FSH ratio.	[70]
Serum biomonitoring and reproductive function	Case/control	110 workers and 113 controls	Petrolchemical factory workers and non-petrochemical workers	0.628 *–0.457 *	The serum BADGE concentrations were sufficiently high to produce hormonal alterations in adult men but didn’t show a statistically significant difference between cases and controls.	[71]
Urinary biomonitoring and semen quality	Case/control	130 cases 88 controls	Workers in factories with and without BPA exposure	1.4 ^c,d^–38.7 ^c,d^	The inverse correlation between increased urine BPA levels and descreased sperm concentration and total count was statistically significant.	[72]
Serum biomonitoring and sex hormones levels	Cross-sectional	33	Workers in factories of epoxy resin production	64.4 ^e^	No association between serum BPA levels and sex hormone levels was noted.	[73]

^a^ g/L, unadjusted urinary BPA; ^b^ geometric mean; ^c^ µg/g, urinary BPA adjusted for creatinine; ^d^ median; ^e^ µg/L, serum BPA; ^f^ µmol/mol creatinine; *^,e^ µg/L, serum BPA/diglycidyl ether (BPA precursor in vivo). BPA: bisphenol A; FT3: free triiodothyronine; FT4: free thyroxin; TT3: total triiodothyronine; TT4: total thyroxin; TSH: thyrotropin; OR: odd ratio; CI: confidence inderval; HBG: hormone binding globulin; PCOS: polycystic ovary syndrome; LH: luteinizing hormone; FSH: follicle stimulating hormone; BADGE: bisphenol A diglycidylether.
